# Validity, Test-Retest Reliability and Long-Term Stability of Magnetometer Free Inertial Sensor Based 3D Joint Kinematics

**DOI:** 10.3390/s18071980

**Published:** 2018-06-21

**Authors:** Wolfgang Teufl, Markus Miezal, Bertram Taetz, Michael Fröhlich, Gabriele Bleser

**Affiliations:** 1 Junior Research Group wearHEALTH, Technische Universität Kaiserslautern, Gottlieb-Daimler-Str. 48, 67663 Kaiserslautern, Germany; miezal@cs.uni-kl.de (M.M.); taetz@informatik.uni-kl.de (B.T.); bleser@informatik.uni-kl.de (G.B.); 2 Department of Sports Science, Technische Universität Kaiserslautern, Erwin-Schrödinger-Str. 57, 67663 Kaiserslautern, Germany; michael.froehlich@sowi.uni-kl.de

**Keywords:** 3D joint kinematics, drift, inertial sensor, soft tissue, test-retest reliability, validity

## Abstract

The present study investigates an algorithm for the calculation of 3D joint angles based on inertial measurement units (IMUs), omitting magnetometer data. Validity, test-retest reliability, and long-term stability are evaluated in reference to an optical motion capture (OMC) system. Twenty-eight healthy subjects performed a 6 min walk test. Three-dimensional joint kinematics of the lower extremity was recorded simultaneously by means of seven IMUs and an OptiTrack OMC system. To evaluate the performance, the root mean squared error (RMSE), mean range of motion error (ROME), coefficient of multiple correlations (CMC), Bland-Altman (BA) analysis, and intraclass correlation coefficient (ICC) were calculated. For all joints, the RMSE was lower than 2.40°, and the ROME was lower than 1.60°. The CMC revealed good to excellent waveform similarity. Reliability was moderate to excellent with ICC values of 0.52–0.99 for all joints. Error measures did not increase over time. When considering soft tissue artefacts, RMSE and ROME increased by an average of 2.2° ± 1.5° and 2.9° ± 1.7°. This study revealed an excellent correspondence of a magnetometer-free IMU system with an OMC system when excluding soft tissue artefacts.

## 1. Introduction

Marker-based optical motion capture (OMC) systems are commonly used in clinical movement analysis [1] and are therefore considered the gold standard. However, despite high resolutions and sub-millimeter accuracy, the application of OMC is expensive, time-consuming, and restricted to a laboratory environment. Therefore, body-worn inertial measurement units (IMUs) present a mobile alternative [1]. IMUs incorporate 3D accelerometers, 3D gyroscopes, and, typically, 3D magnetometers, measuring 3D linear acceleration, 3D angular velocity, and 3D magnetic field, respectively. Using sensor fusion algorithms, e.g., variations of the Kalman filter or optimization based methods [2], it is possible to estimate the IMUs’ orientation in reference to a global coordinate system [3]. Combining more IMUs attached to linked body segments, it is possible to estimate the joint kinematics of the specified segments [1,4,5].

There are drawbacks concerning IMU systems that have to be addressed when measuring human motion. First, IMU-based orientation estimation suffers from drift due to the integration of noisy gyroscope measurements [6]. This is particularly challenging when omitting magnetometer data, which provide a global heading reference and can therefore be used to compensate for drift in the transversal plane [2].

Second, the incorporation of magnetometer measurements is typically based on the assumption of a homogeneous magnetic field, which is often violated [7]. There are efforts to develop methods for handling magnetic disturbances [8,9] or completely omit magnetometer data [4,10,11,12]. Concerning the latter, El-Gohary and McNames [11] present an unscented Kalman filter based approach for estimating the joint angles of a three segment kinematic chain using three IMUs. The kinematic model represents a human arm with stationary shoulder joint position but considers only two rotational degrees of freedom (DOFs) in each joint, including the shoulder, to match the DOFs of the industrial robot arm, which was used for validation in a 15-min trial. For drift reduction, they propose gyroscope and accelerometer bias estimation, limited joint ranges of motion, and zero velocity updates. Seel et al. [4] present a magnetometer-free method for calculating the angles around one dominant axis of one joint using two IMUs. In [4] they consider the knee and ankle flexion/extension angles during walking. The method was evaluated with one transfemoral amputee during repeated 10 m walking trials. Fasel et al. [12] focus on the validation of functional calibration and segment orientation estimation methods adapted for outdoor activities with highly dynamic movements. They recorded and analyzed 120 s of skiing on an indoor skiing carpet with nine IMUs on shanks, thighs, lower back, sternum, upper back, and head. They first estimate the orientation of each IMU separately from the acceleration and angular velocity measurements and then propose a drift correction method for adjacent segments using similar principles as in [4] (i.e., the acceleration vector in the joint position should be identical in the global frame, no matter from which IMU, preceding or following, it was predicted). The drift reduction method is detailed in [13]. The current study investigates a slightly modified version of a previously published sensor fusion algorithm for real-time lower body joint kinematics estimation with seven IMUs on pelvis, thighs, shanks, and feet [14]. In this study, the method was extended with accelerometer bias estimation as in [11], and magnetometer data was completely omitted. In contrast to [11], the method uses a so-called free segments biomechanical model (with six DOFs per segment), which was shown to outperform the kinematic chain model with respect to the influence of model calibration errors and the dependence on undisturbed magnetometer information on simulated and real data from one test person in [2]. In contrast to the magnetometer-free method in [4], the proposed method is not restricted to joint movements with one dominant axis of rotation. In contrast to [12], the segment kinematics are all estimated jointly by fusing the IMU measurements with information from the biomechanical model and environmental constraints (i.e., ground contacts), which results in a built-in drift reduction for the joint angles (cf. Section 3.4 and Section 4.4). Moreover, in contrast to the previously mentioned methods, [14] also provides global segment position estimates, though this study focuses on the estimated orientations.

Researchers use simple IMU set ups to calculate spatio-temporal gait parameters [15,16]. However, the measurement of 3D joint angles based on IMUs is still subject to extensive development and lacks in adequate validity and reliability studies.

Various authors tried to fill this gap [17,18,19,20]. Robert-Lachaine et al. [17] examined a commercially available IMU system, capturing ergonomic lifting and carrying tasks over a time period of 32 min. Zhang and Novak [18] evaluated the IMU derived joint angles of ten subjects during gait. Ferrari et al. [19] compared IMU system based joint kinematics with OMC system derived joint kinematics during a ten meter walk using identical coordinate frames and rigid marker clusters. Al-Amri et al. [20] validated the IMU based joint kinematics of gait, squat, and a jumping task of 27 subjects. Nevertheless, most studies are expandable in terms of the measurement and evaluation protocol. Despite the long-time measurement, Robert-Lachaine et al. [17] did not report results of different time sections of their records. Bergamini et al. [21] examined the global drift of two IMUs attached to the wrist and sacrum during 180 s of level walking. They found a global drift, mainly in the transversal plane.

Other authors used coordinate frames inconsistent between IMU and OMC systems [12,18,20]. Kainz et al. [22] demonstrated the high impact of coordinate system differences on the accuracy of the calculated joint kinematics. Hence, it was a special objective of this study to evaluate the pure technical error between the OMC and IMU system using consistent coordinate frames.

Further, the number of subjects examined rarely exceeds 10 [18,19]. Additionally, most of these studies used marker clusters rigidly fixed to the IMUs for optical joint angle calculation. Thus, both systems suffer from the same amount of soft tissue artefacts (STA), minimizing the error between the systems due to different positioning. Few studies used marker sets attached to anatomical landmarks for the validation rather than marker sets rigidly fixed to the IMUs, therefore taking into account errors due to STA [4,12,20,23]. Al-Amri et al. [20] mainly investigates the reliability of a commercial IMU system, but they did not report detailed results for the validity, especially concerning the frontal and transversal plane joint angles. Nüesch et al. [23] also evaluated a commercial IMU system delivering only sagittal plane joint angles. Seel et al. [4] highlighted the impact of STA on the joint angle data by comparing error measures of the human leg and the prosthesis of one transfemoral amputee. As mentioned above, they also focused on one dominant axis of rotation. In contrast, the present work highlights the amount of errors linked to STA for the complete 3D joint angles of the lower body.

The aim of this study was to fill the mentioned gaps concerning the study design and evaluate a previously published sensor fusion algorithm without using magnetometer information. The main target was to evaluate the validity and test-retest reliability of the estimated 3D joint kinematics. A second objective of the analysis was to examine drift in the estimated kinematics, when measuring over an extended time period (>1 min). Additionally, a specific concern was to highlight the effects of STA on IMU-derived data.

## 2. Materials and Methods

### 2.1. Subjects and Data Acquisition

Twenty-eight healthy subjects (15 females, 13 males; age 24 ± 2.70 years; 70 ± 12.70 kg and 1.76 ± 0.09 m in height) participated in the study. Each of the subjects performed two test sessions on two days (6.75 ± 2.26 days in between). A test session consisted of one static neutral zero position (n-pose) sequence and a 6 min walk test [24]. The study was approved by the ethical committee of the Technische Universität Kaiserslautern (TUK) and meets the criteria of the declaration of Helsinki. After receiving all relevant study information, the participants signed an informed consent to the study including a permission to publish data.

On both test days lower extremity 3D kinematics was simultaneously captured using twelve OptiTrack Prime 13 cameras (NaturalPoint, Inc., Corvallis, OR, USA) and seven XSens MTw Awinda (Xsens Technologies BV, Enschede, The Netherlands) IMUs.

IMUs were activated at least 20 min before measurement start. A static trial was performed before each subject was instrumented, with the sensors lying still for a period of at least 10 s, to estimate and subtract the gyroscope bias. These steps were conducted in accordance with the recommendations of Bergamini et al. [21].

**Figure 1:**
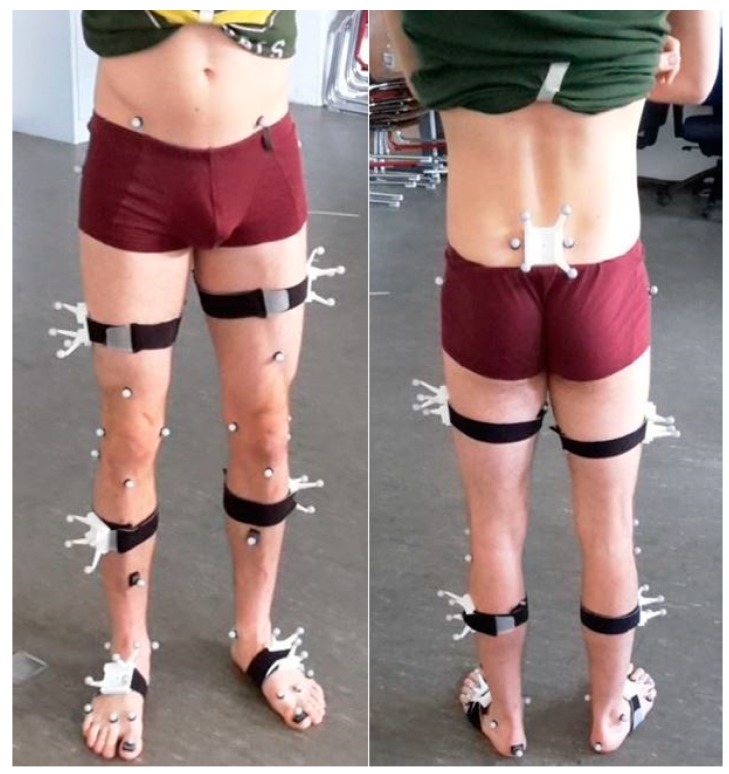
Instrumentation of one exemplary subject. Marker set and rigid marker clusters.

Thirty-two retroreflective markers were attached to anatomical landmarks (AL) according to Leardini et al. [25] and the OptiTrack recommendations. Each IMU was secured in matched 3D printed boxes to which four markers were rigidly attached. These markers were used for unique identification in the optical point cloud as well as for orientation estimation. Using the OptiTrack Software, the origin of the boxes was moved to the center of the attached sensor casing. These box/sensor compounds were fixed to the body segments using straps and double-sided adhesive tape. IMUs were attached on the right and left dorsum of the foot approximately atop the base of metatarsal II-IV, on the right and left lateral aspect of the shank, due to better visibility, on the right and left lateral aspect of the lower third of the thigh and between the Spinae Iliacae Posteriores Superiores approximately atop the sacral base (Figure 1).

Inertial and optical data were simultaneously recorded at 60 Hz using XSens MVN Biomech (Version 4.3.7) and OptiTrack Motive (Version 1.10.0) which were hardware synchronized using a standard 5 V TTL signal. The alignment orientations between the IMUs and the rigid boxes were calculated using the method described in [7]. The biomechanical model according to Cappozzo et al. [26] and the IMU-to-segment calibrations were extracted from the OMC data of the n-pose sequence. The joint centers were also calculated from the OMC data during the n-pose sequence according to the definitions of Visual3D (C-Motion, Inc, Germantown, MD, USA), a widely used software tool for 3D biomechanics research. The first OMC frame of each walking sequence was used as initialization for the IMU-based kinematics estimation. Both systems used the same biomechanical model.

The inertial data was processed with an iterated extended Kalman filter (IEKF) approach based on [14] while omitting magnetometer information. The gyroscope biases were extracted from a static sequence (see above), while the accelerometer biases were estimated in the IEKF along with the kinematics estimation using the model described in [11,27]. The same sequence was processed twice: initially to obtain a converged estimate of the acceleration bias, which was then used as initial guess in the second run. The estimated segment orientations were used to derive relative joint orientations. These were decomposed into joint angles using Euler angle decomposition [28]. The sensor fusion method is detailed in Appendix A.

To minimize STA, the OMC-based joint angles were derived from marker clusters on the rigid boxes (condition 1). For secondary analyses the joint angles were calculated based on the markers attached to the anatomical landmarks (condition 2). Initial contact (IC) was detected based on the left and right heel marker [29]. Turning phases in the 6 min walk tests were omitted. In order to investigate drift behavior, 10 left and right steps (one trial) were identified at three sections, i.e., beginning (A), middle (B), and end (C) of the test. All joint angle curves were normalized to 100 percent gait cycle (GC).

### 2.2. Statistical Analysis

To evaluate the IMU system, the root mean squared error (RMSE) and range of motion error (ROME), as well as 95% confidence interval (CI) were calculated for hip, knee, ankle joint, and the global pelvis orientation per section per GC. Further, Bland-Altman analysis (BA) was conducted to evaluate the limits of agreement (LoA) between the mean joint angle waveforms over all 28 subjects for both systems, considering only the normalized GC of section A. The results of the BA analysis are presented in the form 0.0°–0.0° ± 0.0°–0.0°. The first two numbers indicate the minimum and maximum of the mean differences between the systems. The last two numbers indicate the minimum and maximum of the limits of agreement (95% CI) of the two systems. The coefficient of multiple correlation (CMC) was calculated for each parameter per section per GC according to Ferrari et al. [30]. In [30] they showed that if a joint angle waveform reveals a similar ROM compared to the overall offset between the two signals, the CMC can become a complex number. If that happened in the current calculations for individual subjects, these results were not considered for further analysis. All these calculations were conducted for both, condition 1 and condition 2.

A paired *t*-test was performed to identify significant differences in the RMSE and ROME of the joint angles of all sections between condition 1 and condition 2. Alpha level was set a priori to 0.05. The Chi-square goodness-of-fit test was carried out to check for normal distribution in the data.

For the evaluation of the test-retest reliability, the intraclass correlation coefficient (ICC) for inter-day reliability was calculated for test day one and test day two for both systems for every joint and section according to McGraw and Wong [31]. CMC and ICC values were rated according to Koo and Li [32].

The global heading direction error of the pelvis in the transversal plane (pelvis rotation error) was examined at minute 0, 1, 2, 3, 4, 5, and 6. In addition, the RMSE and the ROME of the joint angles and pelvis flexion/obliquity were examined regarding potential linear trends over time. Therefore, lines were fitted via linear least squares regression to the RMSE and ROME values of each GC for the abovementioned angles and all test persons (Matlab function “fit” with “fittype = poly1”). The slopes of the fitted lines were computed and plotted to evaluate potential trends. Processing of the joint angles and statistics were conducted in Matlab 2015 (Mathworks Inc., Natick, MA, USA).

## 3. Results

### 3.1. Condition 1—Marker Clusters

**Figure 2:**
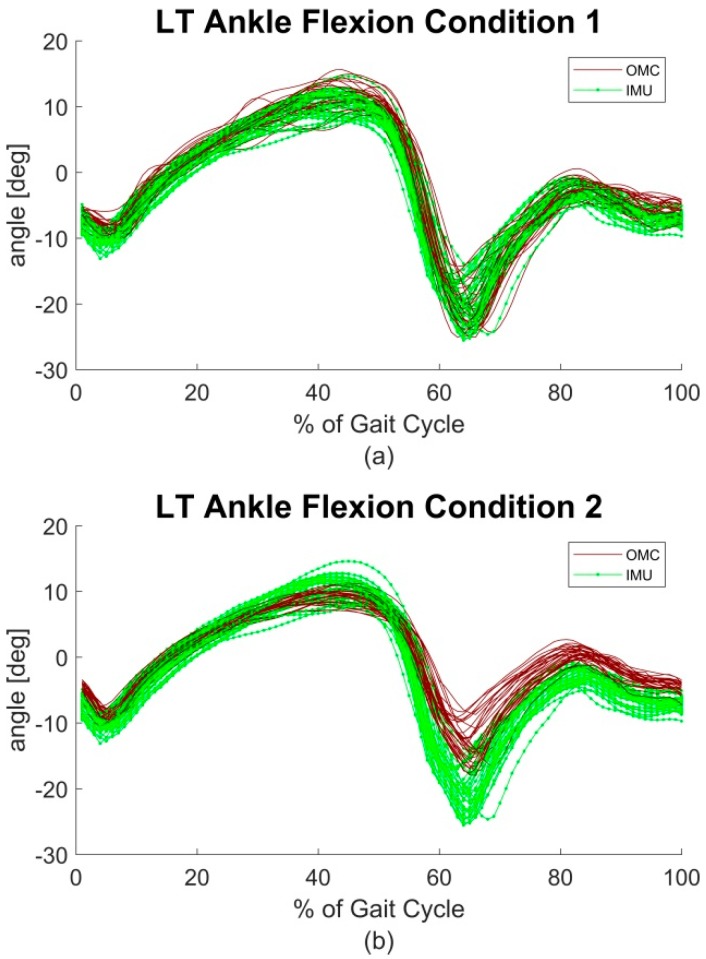
Left (LT) ankle flexion of a representative subject. Soft tissue artefacts (STA) error excluded (**a**) and included (**b**). At 60 to 100% Gait Cycle (GC) appears a typical offset between optical motion capture (OMC) and inertial measurement unit (IMU)-derived data.

RMSE and ROME for all parameters over all sections are shown in Table 1. RMSE and ROME between the two systems revealed mean values lower than 2.40° and lower than 1.60° respectively in all joints. The poorest outcome concerning the RMSE was evident in knee rotation (1.75°–2.34°) and knee abduction for ROME (1.11°–1.58°). Figure 2a exemplary shows the left (LT) ankle flexion of a representative subject.

**Figure 3:**
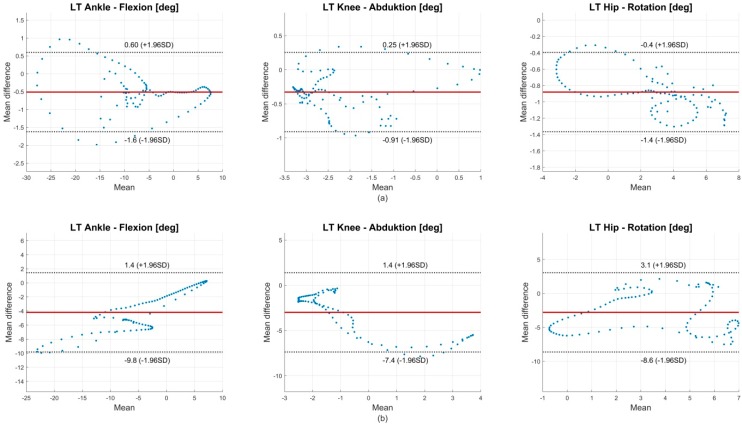
Bland-Altman (BA) diagrams for the most affected joint angles of every plane. The plots show the agreement between the 100% GC normalized joint angle waveforms of OMC and IMU system (averaged over all 28 participants). A normalized joint angle waveform contains 100 data points, which results in 100 data points in the BA diagrams. Upper row (**a**) shows condition 1, the lower row (**b**) condition 2. The solid line indicates the mean difference. The dashed lines indicate the limits of agreement (LoA) (95% CI of the mean difference).

**Figure A2:**
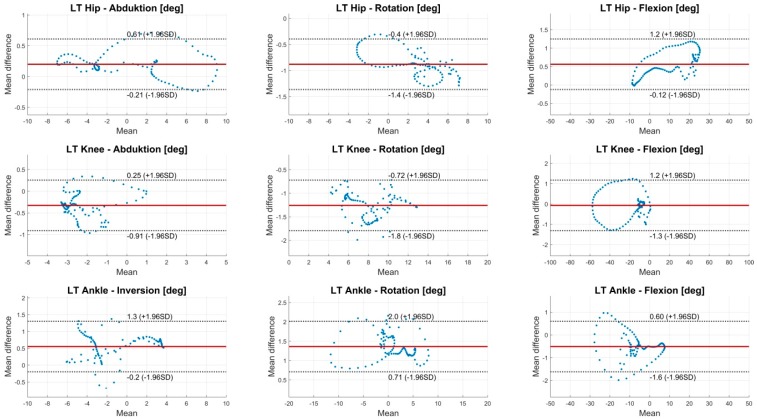
BA diagrams of 3D joint rotations of hip, knee and ankle of the left side for condition 1. The solid line indicates the mean difference. The dashed lines indicate the LoA (95% CI of the mean difference).

**Figure A4:**
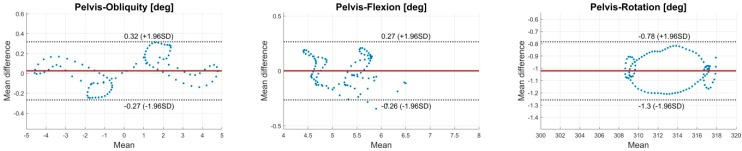
BA diagrams of 3D joint rotations of the pelvis for condition 1. The solid line indicates the mean difference. The dashed lines indicate the LoA (95% CI of the mean difference).

BA analysis revealed mean differences between the systems of −0.6°–0.6° ± 0.5°–1.3° for sagittal joint angles. The best outcome showed the global pelvis flexion with a bias 0.0° ± 0.3°. Frontal plane joint angles showed biases of −0.3°–0.1° ± 0.3°–0.8° and transversal plane joint angles revealed biases of −0.9°–1.4° ± 0.3°–0.7°. BA plots are shown in Appendix B (Figure A2 through Figure A4). Figure 3 (upper row) shows the BA diagrams for the most affected joint angles of every plane.

**Figure 4:**
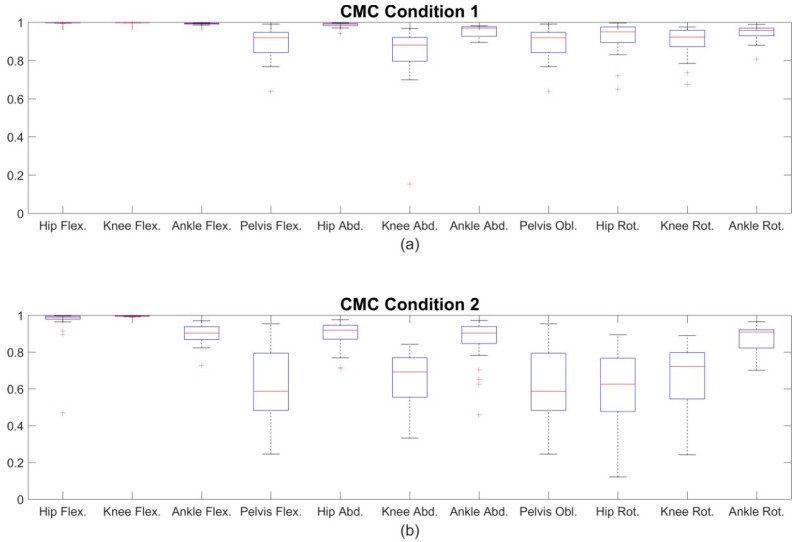
Mean coefficient of multiple correlation (CMC) values over all subjects of section A for condition 1 (**a**) and condition 2 (**b**). Exemplary only the joint angles of the left lower extremity are shown.

The CMC showed very high waveform similarity for the joint angles in the sagittal plane with mean values ranging from 0.99 to 1. Concerning the frontal and transversal plane, the CMC showed slightly lower correspondence with mean values ranging from 0.88 to 0.99. All CMC values are mapped in the box-and-whisker-plot in Figure 4a.

### 3.2. Condition 2—Skin Markers

Most error measures showed results inferior to condition 1, when considering STA.

RMSE and ROME for all joint angles over all sections are shown in Table 2. RMSE showed values lower than 6.00° over all planes and joints. ROME showed values lower than 6.10° for hip, knee and pelvis in all planes, and ankle joint in transversal and frontal plane. However, the movement in the sagittal plane in the ankle joint revealed a ROME of up to 10.66°. Figure 2b exemplary shows the LT ankle flexion of a representative subject for condition 2.

**Figure A5:**
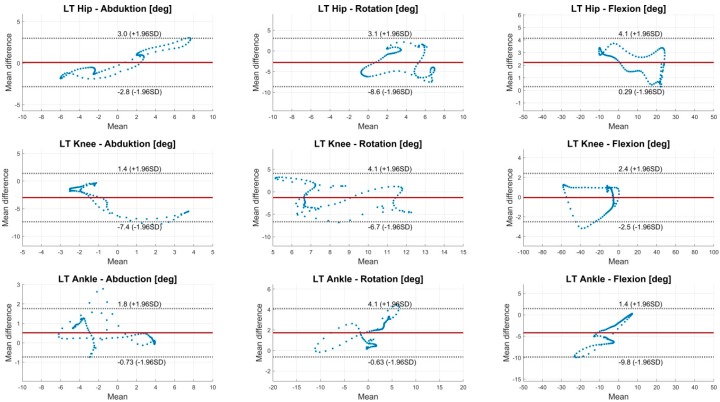
BA diagrams of 3D joint rotations of hip, knee, and ankle of the left side for condition 2. The solid line indicates the mean difference. The dashed lines indicate the LoA (95% CI of the mean difference).

**Figure A6:**
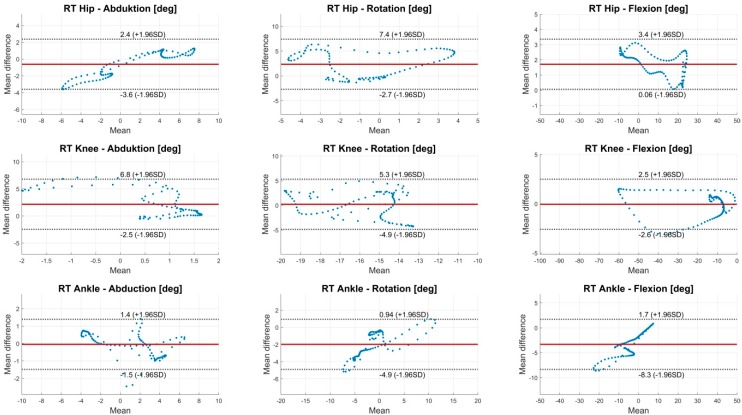
BA diagrams of 3D joint rotations of hip, knee, and ankle of the right side for condition 2. The solid line indicates the mean difference. The dashed lines indicate the LoA (95% CI) of the mean difference.

Concerning condition 2 BA analysis revealed mean differences inferior to condition 1. Sagittal joint angles showed biases of −4.2°–2.2° ± 1.6°–5.6°. Frontal angles showed biases of −3.0°–1.0° ± 0.8°–4.5°. Transversal angles showed biases of −2.8°–2.4° ± 2.4°–5.8°. BA plots are shown in Appendix B (Figure A5 through Figure A6). Figure 3 (lower row) shows the BA diagrams for the most affected joint angles of every plane.

CMC values in the sagittal plane were good to excellent with values ranging from 0.89 to 1. CMC values of the transversal and frontal plane showed moderate to good results ranging from 0.53 to 0.90. The hip joint in the transversal plane exhibited the poorest outcome (CMC = 0.53 – 0.67). All CMC values are mapped in the box-and-whisker-plot in Figure 4b.

The paired *t*-test revealed significant differences between condition 1 and condition 2 of the RMSE in all joint angles and sections. ROME showed significant differences between condition 1 and 2 in all joint angles and sections excepting the right hip flexion of section A (*p* = 0.08). For detailed results see Table 3.

### 3.3. Test-Retest Reliability

The ICC revealed moderate to excellent correlations over all joints in the frontal and transversal plane (0.52–0.93) and excellent values in the sagittal plane (0.94–0.99) (Table 4). The knee joint in the frontal and transversal plane, the pelvis in the frontal plane, and the hip joint in the transversal plane showed the lowest values (0.52–0.76). This tendency consented with the results from ICC calculation of the optical system (0.63–0.83). However, overall ICC values of the OMC system (0.63–0.99) were higher compared to the IMU system.

### 3.4. Drift

**Figure 5:**
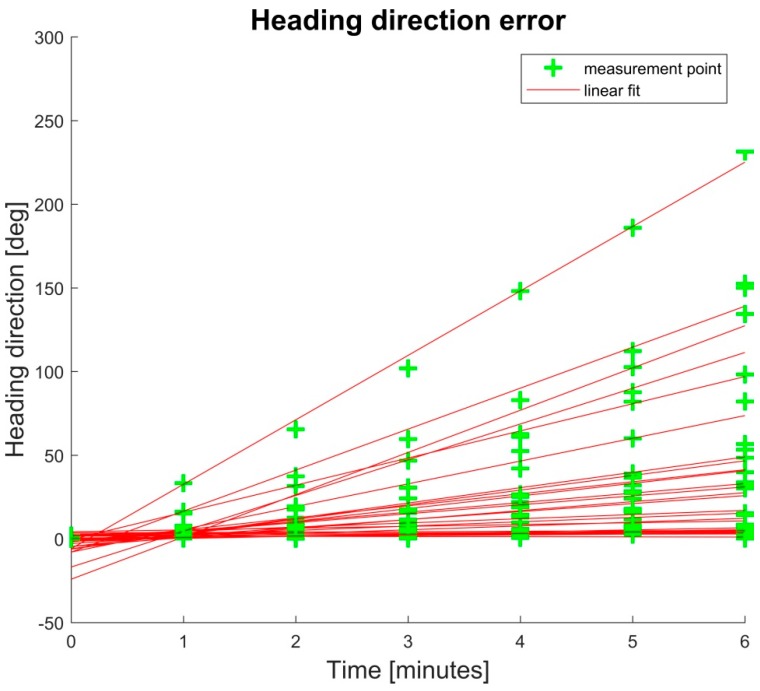
Error of the global pelvis rotation in the transversal plane at minute 0, 1, 2, 3, 4, 5, and 6 for all subjects. The drift in the global transversal plane showed a linear trend but no consistent dimension.

The global heading direction of the pelvis in the transversal plane (pelvis rotation) drifted linearly but not consistently between subjects (45° ± 58°). Global heading errors after six minutes ranged from 0.21° up to 230° (Figure 5).

**Figure 6:**
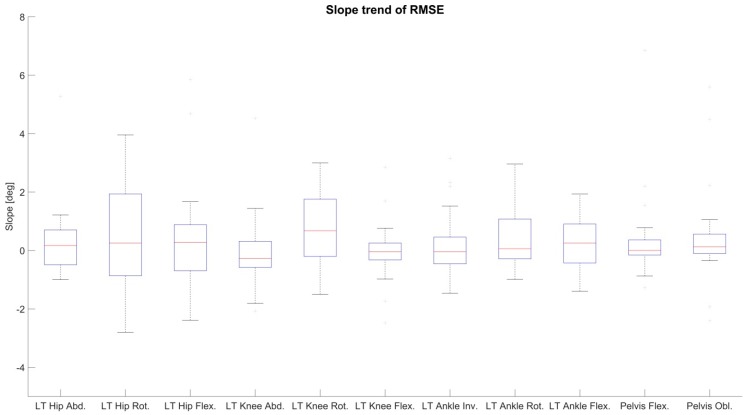
Evaluation of linear regression of RMSE for condition 2 over time exemplary for the left lower extremity and global pelvis angles in sagittal and frontal plane. On the vertical axis is the slope of the regression line in degree. Median slope values, quartiles, and whiskers are above and below zero indicating no increasing trend of RMSE over time.

**Figure 7:**
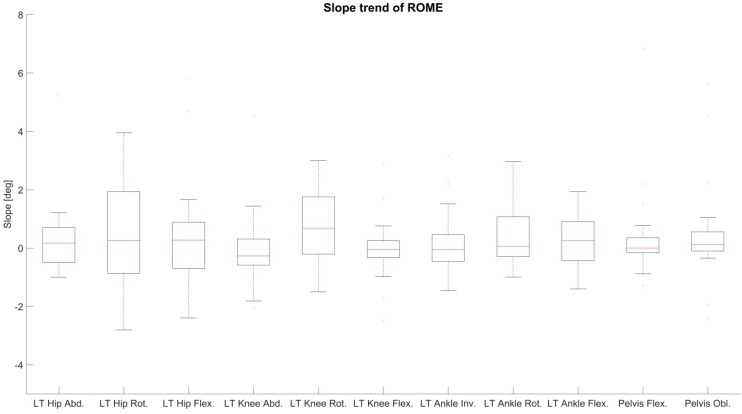
Evaluation of linear regression of ROME for condition 2 over time exemplary for the left lower extremity and global pelvis angles in sagittal and frontal plane. On the vertical axis is the slope of the regression line in degree. Median slope values, quartiles as well as whiskers are above and below zero indicating no increasing trend of ROME over time.

To analyze the drift in the joint angle data, changes in the RMSE and ROME of condition 2 over time were evaluated through linear least squares regression (line fitting). The slopes of the fitted lines of RMSE and ROME for all joint angles of the left side and global pelvis flexion and obliquity of all test persons are shown in Figure 6 and Figure 7. Consistent positive slopes (i.e., increasing RMSE/ROME error values over time) would indicate a systematic drift over time. However, as visible in the figures, the slopes of both RMSE and ROME over time reside above as well as below zero, so that there is no clear trend over all test persons visible. Moreover, the slopes are in a range where the errors cannot be distinguished from noise given disturbing effects such as STA.

## 4. Discussion

This paper evaluated the performance of a sensor fusion algorithm for estimating 3D kinematics from gyroscope and accelerometer data.

### 4.1. Condition 1—Marker Clusters

RMSE in the present examination outperformed the results of Robert-Lachaine et al. [17] (1.90°–7.30°). Ferrari et al. [19] showed similar results for their “Off” calculation (−2°–+2°) and for ROME (0.60°–1.50°).

The BA analysis revealed excellent agreement between the systems. Mean difference results were similar to the results of Robert-Lachaine et al. [17]. They consent with the present findings of the sagittal joint angles showing the best agreement and the transversal joint angles the poorest agreement between the two systems. However, the limits found by Robert-Lachaine et al. [17] ranged from 2.8°–7.0°, compared to limits of 0.3°–1.3° in the present findings.

CMC values showed excellent correspondence between the systems in the sagittal plane and good to excellent correspondence in the frontal and transversal plane. These findings are in accordance with Ferrari et al. [19] and outperform the results of Zhang et al. [18]. However, the former performed an offset correction to increase waveform similarity and did not consider transversal and frontal plane of the knee joint. Zhang et al. used different coordinate systems for joint angle calculation [18]. The findings of Robert-Lachaine et al. [17] showed better CMC values concerning knee rotation and abduction (0.91 and 0.97). For ankle joint rotation and abduction, they report their lowest CMC values (0.89 and 0.77). In this case, the current system achieved higher correspondence (0.96–0.98). However, Robert-Lachaine et al. [17] do not report whether these results are mean values over the entire time period of 32 min. Furthermore, Robert-Lachaine et al. [17] analyzed a complex combination of movements rather than a standardized motion.

CMC values were lowest for knee abduction (0.88–0.93). The majority of literature reports lowest CMC outcomes considering motion in the transversal and frontal planes [17,18,19]. However, during walking, movements in these planes show smaller ranges of motion compared to the sagittal plane. It has been shown that the CMC decreases as the amplitude of movement decreases [33]. This might also explain the rather good results of Robert-Lachaine et al. concerning the CMC of the knee in frontal and transversal plane (0.91–0.97). In this study, subjects had to perform lifting and turning tasks which could have led to increased ROM in the mentioned planes.

### 4.2. Condition 2—Skin Markers

As mentioned, most values of errors increased. This consented with the findings of Seel et al. [4] who compared knee and ankle sagittal joint kinematics of the human leg and the transfemoral prosthesis of an above-knee amputee. They found the errors on the human leg four times higher than on the prosthesis. On average, RMSE and ROME increased by 2.20° ± 1.50° and 2.90° ± 1.70°, respectively. Nüesch et al. [23] reported RMSE of the sagittal plane for hip, knee, and ankle joint (9.60°, 7.60°, 4.50°). The present system revealed better results concerning the sagittal plane for hip and knee (3.83°, 2.66°) and similar results for the ankle (5.48°). However, Nüesch et al. [23] conducted their examination on a treadmill, which could have affected IMU-derived data [14]. Fasel et al. [12] found in their evaluation of skiing similar hip abduction ROME (−3.3° ± 4.1°), slightly higher knee abduction ROME (4.2° ± 5.5°) and distinctively higher hip flexion ROME (−10.7° ± 4.3°). Their findings concerning knee flexion, hip and knee rotation showed better results for ROME around −0.1° and 0.5°. However, note that Fasel et al. [12] investigated a different task, which limits comparability. Further, they conducted their examination on an indoor skiing carpet, comparable to a treadmill, therefore inviting the same considerations associated with Nüesch et al. [23]. Interestingly, ROME of the ankle joint flexion increased from 1.61° in condition 1 to 10.66° in condition 2. In the IMU system and when calculating joint angles based on marker clusters, the foot is assumed to be one rigid segment. However, the foot is a complex organization of bony segments [34]. Skin markers attached to anatomical landmarks on the foot are in fact placed on different segments rather than on one segment only. This might explain the differences in the ankle flexion between IMU and OMC system.

BA analysis also showed increased biases compared to condition 1. The ankle flexion was the most affected joint angle in the sagittal plane, the knee abduction the most affected joint angle in the frontal plane and the hip rotation the most affected joint angle in the transversal plane (Figure 3). CMC values decreased mostly for the transversal and frontal plane. This might be due to the increased uncertainty concerning CMC and smaller ranges of motion [33]. However, the ankle joint flexion still presents good to excellent values. Qualitative examination of the ankle flexion waveforms showed that there is an excellent match between waveforms of the IMU system and the OMC system at about 10 to 50% GC. However, at 60 to 100% GC an offset appeared (Figure 2b). This might explain the difference of ROM, but still similar shape of waveform and good CMC values. Al-Amri et al. [20] showed a similar shape of waveform for ankle flexion in their examination. No such differences were found between the IMU system and the OMC system based on the marker clusters (Figure 2a). Al-Amri et al. [20] examined in their recent study the validity of a commercial IMU system during one 8 m walk, taking into account errors due to STA. They found excellent CMC values concerning the sagittal plane of all three joints and the frontal plane of the hip joint but stated a poor outcome for the remaining joints in the frontal and transversal plane. However, they did not report exact values of the CMC for the frontal and transversal plane because results were complex numbers at times. In this study, CMC values for some subjects also resulted in complex numbers. On average, 3 out of 28 subjects per joint angle resulted in complex numbers. However, these subjects were ignored for the calculation of the mean CMC values shown in Figure 4. Nevertheless, concerning the findings of Al-Amri et al. [20], R² values indicated rather poor correlations for the joint angles in the transversal plane. That consents with the present results, showing moderate CMC values in the hip joint angle of the transversal plane (Figure 4b). Al-Amri et al. [20] used different biomechanical models for their analysis and stated possible uncertainties in the optical data due to the marker protocol. Further, Fiorentino et al. [35] showed that the 3D hip joint angles and ROM based on OMC systems are significantly influenced by STA.

### 4.3. Test-Retest Reliability

ICC values revealed moderate to excellent results for all joint angles. Knee abduction (0.56–0.58) and pelvis obliquity (0.52) showed the lowest test-retest correlation. These results are in accordance with Mills et al. [36]. However, the ICC calculation for the OMC system based on skin markers showed overall better outcome. This fact might be explained by expert marker placement. The IMUs were attached only approximately to the same spot on the segments. Furthermore, the IMUs were more prone to STA than the skin markers due to the rigid boxes and positioning focused on better visibility. These circumstances may have caused different amounts of errors on the two measurement days. For both systems, values of measures of reproducibility were not high. A possible explanation is the fact that inter-day variability of gait is considered higher than, for example, intra-day variability [36].

### 4.4. Drift

One difference of the current IMU system compared to the systems used in the referenced studies is the omission of the magnetometer information. Favre et al. [10] measured the 3D knee angle omitting magnetometer information and presented results with a mean error of 4.00° to 8.10°. They also introduced a functional calibration method to align the joint coordinate system. Note that the present evaluation relates to the sensor fusion algorithm, while the calibration, as mentioned in Section 2.1, was obtained from the OMC system. Thus, the results presented in this study can be considered free of calibration errors.

However, omitting global heading direction information (obtained through undisturbed magnetometer measurements) typically leads to drift [2,12]. In the present study, gait was measured for six minutes. Robert-Lachaine et al. [17] analyzed ergonomic tasks over a period of 32 min. However, they do not state results for different sections of their test procedure and thus give no hint as to whether drift appeared in the kinematic data or not.

Fasel et al. [12] measured skiing for 120 s. They reported errors in their joint angle results (based on individual per-IMU orientation estimates) due to drift and introduced methods for drift reduction [13] based on adjacent segments as a second processing stage (cf. Section 1). On the contrary, the present study revealed no systematic drift over all test persons, neither in the 3D joint angle data nor in the global pelvis flexion/obliquity. This is due to including biomechanical constraints in terms of connected segments and environmental constraints in terms of ground contacts directly into the estimation. However, Fasel et al. [13] reported that mainly highly flexed joint angles were affected from the drift. In their examination of skiing subjects reached distinctively higher peaks in hip and knee flexion compared to the present study.

Bergamini et al. [21] examined the drift in the orientation obtained from two inertial sensors mounted on wrist and pelvis during 180 s of gait. In the transversal plane, a drift of up to 40° was measured. In the sagittal plane and frontal plane, drift was smaller with values up to approximately 5°. The findings of Bergamini et al. [21] are comparable with this study’s results for drift in the global heading direction estimate (investigated at the root segment, i.e., the pelvic rotation) measured at values of up to 230°. Note, inter-segment constraints do not provide corrective information concerning the global heading direction, which explains the linear drift observed. Consequently, the global pelvic rotation was neglected in the evaluation. However, the evaluation and interpretation of the ROM of the pelvic rotation and its reproducibility were independent of the drift.

## 5. Conclusions

The present algorithm for the calculation of 3D joint angles based on gyroscope and accelerometer data from seven IMUs mounted on the lower body shows good to excellent agreement when compared to a common OMC system and excluding STA. However, in this study, the influence of STA was shown. Especially the ankle joint was highly affected by these and further artefacts, e.g., a limited biomechanical model. Further research has to be conducted to better compensate these effects.

In terms of reliability, the results indicate that an OMC system combined with an experienced examiner delivers a better outcome, particularly for knee abduction and rotation and the ankle joint. Better placement of the shank sensor and smaller IMUs might improve overall reliability and sensitivity to STA. 

Systematic drift was observed only in the global transversal plane angle (investigated at the root segment, i.e., the pelvis rotation). There was no systematic drift observed over all test persons in the other kinematic parameters. However, in clinical gait analysis the ROM per GC is the more essential criterion, which was also measured with satisfying accuracy for the pelvis rotation.

The current sensor fusion algorithm was not only shown to be comparable to other algorithms, but also tends to outperform most algorithms examined so far in terms of its accuracy while being magnetometer-independent. However, it has to be considered that this study focused on the evaluation of the sensor fusion algorithm, while the IMU-to-segment calibration, the biomechanical model and the initialization were obtained from the OMC system. Therefore, the next step consists of evaluating the validated sensor fusion method in a setup, where all information was obtained purely from the IMU system.

Nevertheless, this examination reveals promising results of a magnetometer–independent sensor fusion algorithm that showed no systematic drift in the joint angle data. Therefore, a stand-alone system incorporating this algorithm provides potential for applications in clinical gait analysis and further implementations.

## Figures and Tables

**Table 1:** Mean root mean squared error (RMSE) and mean range of motion error (ROME) of condition 1 over all subjects ± standard deviation (SD); brackets contain 95% confidence interval (CI). A, B, C indicate beginning, middle, and end of the 6 min walk test.

	RMSE (deg) ± SD (95% CI)			ROME (deg) ± SD (95% CI)		
	A	B	C	A	B	C
LT Hip—Abduction	1.05 ± 0.42 (0.78–1.11)	1.14 ± 0.55 (0.75–1.17)	1.06 ± 0.45 (0.77–1.12)	0.54 ± 0.21 (0.43–0.59)	0.57 ± 0.29 (0.38–0.60)	0.57 ± 0.27 (0.44–0.64)
LT Hip—Rotation	1.94 ± 0.92 (1.49–2.20)	2.29 ± 1.36 (1.85–2.91)	2.25 ± 1.16 (1.80–2.70)	0.68 ± 0.27 (0.53–0.74)	0.70 ± 0.28 (0.55–0.77)	0.68 ± 0.28 (0.56–0.75)
LT Hip—Flexion	1.02 ± 0.35 (0.79–1.06)	0.99 ± 0.29 (0.83–1.06)	1.00 ± 0.32 (0.78–1.02)	0.93 ± 0.36 (0.71–1.00)	0.89 ± 0.36 (0.72–0.99)	0.85 ± 0.37 (0.70–0.99)
LT Knee—Abduction	1.59 ± 0.48 (1.22–1.59)	1.58 ± 0.50 (1.26–1.65)	1.57 ± 0.51 (1.31–1.71)	1.58 ± 0.79 (1.20–1.81)	1.54 ± 0.92 (0.97–1.68)	1.54 ± 0.83 (1.09–1.73)
LT Knee—Rotation	2.34 ± 1.08 (1.63–2.48)	2.34 ± 1.16 (1.43–2.33)	2.27 ± 1.10 (1.37–2.23)	1.09 ± 0.32 (0.92–1.16)	1.09 ± 0.39 (0.93–1.23)	1.16 ± 0.41 (0.98–1.30)
LT Knee—Flexion	1.47 ± 0.34 (1.25–1.51)	1.44 ± 0.31 (1.29–1.53)	1.41 ± 0.34 (1.17–1.44)	0.70 ± 0.27 (0.57–0.78)	0.67 ± 0.27 (0.51–0.72)	0.72 ± 0.33 (0.60–0.86)
LT Ankle—Abduction	1.61 ± 0.39 (1.42–1.73)	1.63 ± 0.36 (1.50–1.78)	1.62 ± 0.43 (1.35–1.68)	1.29 ± 0.51 (0.96–1.35)	1.43 ± 0.43 (1.29–1.62)	1.22 ± 0.39 (0.97–1.27)
LT Ankle—Rotation	2.16 ± 0.68 (1.80–2.33)	2.12 ± 0.65 (1.70–2.21)	2.13 ± 0.68 (1.69–2.19)	1.56 ± 0.57 (1.18–1.63)	1.51 ± 0.61 (1.13–1.59)	1.53 ± 0.45 (1.35–1.69)
LT Ankle—Flexion	1.55 ± 0.34 (1.46–1.72)	1.54 ± 0.36 (1.41–1.69)	1.61 ± 0.47 (1.35–1.72)	0.97 ± 0.38 (0.73–1.03)	0.98 ± 0.38 (0.73–1.02)	1.08 ± 0.44 (0.85–1.19)
RT Hip—Abduction	1.09 ± 0.54 (0.63–1.05)	1.09 ± 0.55 (0.68–1.11)	1.12 ± 0.54 (0.69–1.11)	0.56 ± 0.22 (0.42–0.59)	0.55 ± 0.26 (0.32–0.52)	0.53 ± 0.25 (0.38–0.57)
RT Hip—Rotation	1.64 ± 1.00 (1.00–1.77)	1.78 ± 1.76 (0.68–2.04)	2.07 ± 1.72 (0.92–2.25)	0.65 ± 0.47 (0.40–0.76)	0.56 ± 0.19 (0.46–0.60)	0.51 ± 0.20 (0.42–0.57)
RT Hip—Flexion	0.98 ± 0.51 (0.68–1.07)	0.89 ± 0.30 (0.68–0.91)	0.86 ± 0.28 (0.69–0.91)	0.98 ± 1.26 (0.21–1.18)	0.73 ± 0.40 (0.52–0.83)	0.69 ± 0.43 (0.44–0.77)
RT Knee—Abduction	1.26 ± 0.51 (0.90–1.30)	1.26 ± 0.44 (1.08–1.43)	1.24 ± 0.48 (0.90–1.27)	1.11 ± 0.54 (0.79–1.21)	1.12 ± 0.59 (0.77–1.23)	1.19 ± 0.70 (0.69–1.23)
RT Knee—Rotation	1.75 ± 0.63 (1.38–1.87)	1.91 ± 0.72 (1.38–1.93)	1.93 ± 0.84 (1.49–2.14)	1.03 ± 0.57 (0.65–1.09)	0.90 ± 0.42 (0.67–1.00)	1.00 ± 0.45 (0.69–1.04)
RT Knee—Flexion	1.51 ± 0.43 (1.31–1.64)	1.40 ± 0.28 (1.28–1.50)	1.37 ± 0.27 (1.26–1.47)	0.76 ± 0.41 (0.43–0.75)	0.75 ± 0.30 (0.56–0.79)	0.71 ± 0.31 (0.47–0.71)
RT Ankle—Abduktion	1.33 ± 0.35 (1.09–1.36)	1.27 ± 0.33 (1.07–1.33)	1.30 ± 0.29 (1.13–1.35)	1.02 ± 0.48 (0.70–1.07)	1.08 ± 0.49 (0.79–1.06)	0.97 ± 0.35 (0.79–1.06)
RT Ankle—Rotation	1.52 ± 0.41 (1.27–1.59)	1.56 ± 0.46 (1.26–1.62)	1.63 ± 0.51 (1.29–1.68)	1.27 ± 0.57 (0.90–1.34)	1.18 ± 0.48 (0.89–1.27)	1.18 ± 0.48 (0.92–1.29)
RT Ankle—Flexion	1.60 ± 0.36 (1.43–1.71)	1.60 ± 0.38 (1.44–1.74)	1.60 ± 0.42 (1.32–1.65)	1.02 ± 0.37 (0.78–1.07)	0.97 ± 0.38 (0.78–1.07)	0.91 ± 0.38 (0.68–0.97)
Pelvis—Flexion	0.64 ± 0.18 (0.55–0.69)	0.62 ± 0.21 (0.52–0.68)	0.62 ± 0.21 (0.51–0.67)	0.32 ± 0.15 (0.22–0.34)	0.35 ± 0.20 (0.25–0.40)	0.33 ± 0.20 (0.25–0.41)
Pelvis—Obliquity	0.62 ± 0.16 (0.57–0.69)	0.61 ± 0.20 (0.51–0.67)	0.59 ± 0.18 (0.47–0.61)	0.31 ± 0.11 (0.23–0.32)	0.32 ± 0.12 (0.24–0.33)	0.33 ± 0.10 (0.28–0.36)
Pelvis—Rotation	x	x	x	0.42 ± 0.15 (0.32–0.43)	0.47 ± 0.22 (0.35–0.52)	0.51 ± 0.29 (0.29–0.51)

**Table 2:** Mean RMSE and ROME of condition 2 over all subjects ± SD; brackets contain 95% CI. A, B, C indicate beginning, middle and end of the 6 min walk test.

	RMSE (deg) ± SD (95% CI)			ROME (deg) ± SD (95% CI)		
	A	B	C	A	B	C
LT Hip—Abduction	2.57 ± 0.88 (2.14–2.83)	2.69 ± 1.05 (2.11–2.92)	2.69 ± 1.03 (2.05–2.85)	4.91 ± 2.14 (3.74–5.40)	4.85 ± 2.24 (3.84–5.57)	4.94 ± 2.14 (3.93–5.58)
LT Hip—Rotation	5.37 ± 1.66 (4.36–5.64)	5.60 ± 2.16 (4.52–6.20)	5.54 ± 2.10 (4.37–6.00)	3.98 ± 2.63 (2.48–4.52)	4.17 ± 2.61 (3.27–5.29)	4.24 ± 2.82 (3.12–5.31)
LT Hip—Flexion	3.53 ± 3.37 (1.25–3.87)	3.64 ± 3.47 (1.39–4.08)	3.67 ± 3.53 (1.26–4.00)	1.67 ± 1.22 (0.88–1.82)	1.42 ± 0.94 (0.76–1.50)	1.42 ± 0.96 (0.71–1.45)
LT Knee—Abduction	4.19 ± 1.15 (3.63–4.53)	4.14 ± 1.22 (3.53–4.48)	4.13 ± 1.20 (3.45–4.38)	2.89 ± 1.74 (1.83–3.18)	2.76 ± 1.93 (1.68–3.18)	2.85 ± 1.98 (1.83–3.37)
LT Knee—Rotation	4.56 ± 1.33 (3.80–4.83)	4.70 ± 1.40 (4.02–5.11)	4.72 ± 1.44 (4.02–5.13)	3.53 ± 2.08 (2.11–3.72)	3.78 ± 2.05 (2.43–4.02)	3.69 ± 2.33 (2.00–3.81)
LT Knee—Flexion	2.38 ± 0.63 (2.16–2.64)	2.38 ± 0.61 (2.03–2.50)	2.40 ± 0.64 (2.05–2.55)	1.48 ± 1.07 (0.78–1.62)	1.58 ± 1.15 (0.87–1.76)	1.59 ± 1.14 (0.95–1.84)
LT Ankle—Abduction	2.92 ± 1.31 (1.93–2.95)	3.01 ± 1.41 (1.97–3.06)	3.00 ± 1.37 (1.91–2.97)	2.49 ± 1.40 (1.80–2.88)	2.52 ± 1.61 (1.32–2.57)	2.53 ± 1.47 (1.54–2.68)
LT Ankle—Rotation	3.28 ± 1.32 (2.38–3.41)	3.41 ± 1.37 (2.84–3.91)	3.45 ± 1.32 (2.85–3.87)	4.74 ± 2.25 (3.90–5.65)	5.02 ± 2.52 (3.75–5.70)	4.94 ± 2.56 (3.59–5.57)
LT Ankle—Flexion	5.30 ± 1.56 (4.52–5.73)	5.42 ± 1.61 (4.55–5.79)	5.48 ± 1.65 (4.60–5.88)	10.07 ± 2.18 (8.94–10.63)	10.63 ± 2.50 (9.51–11.44)	10.66 ± 2.65 (9.62–11.68)
RT Hip—Abduction	2.58 ± 0.64 (2.35–2.85)	2.62 ± 0.63 (2.34–2.83)	2.63 ± 0.65 (2.47–2.98)	4.80 ± 1.44 (4.41–5.53)	4.71 ± 1.48 (4.15–5.30)	4.68 ± 1.53 (4.05–5.24)
RT Hip—Rotation	5.01 ± 1.37 (4.44–5.51)	4.97 ± 1.26 (4.20–5.18)	5.01 ± 1.07 (4.60–5.43)	3.01 ± 1.83 (1.77–3.19)	2.93 ± 1.54 (1.98–3.17)	3.12 ± 1.50 (2.15–3.31)
RT Hip—Flexion	3.57 ± 3.23 (1.27–3.77)	3.76 ± 3.34 (1.54–4.13)	3.83 ± 3.33 (1.61–4.19)	1.48 ± 0.62 (1.00–1.49)	1.52 ± 0.77 (1.00–1.59)	1.53 ± 0.86 (1.10–1.76)
RT Knee—Abduction	3.83 ± 1.72 (2.52–3.85)	3.79 ± 1.69 (2.53–3.84)	3.72 ± 1.68 (2.45–3.75)	3.16 ± 1.66 (2.07–3.35)	3.21 ± 1.77 (2.27–3.65)	3.21 ± 1.86 (2.22–3.66)
RT Knee—Rotation	4.41 ± 1.01 (3.76–4.54)	4.48 ± 1.06 (3.71–4.53)	4.54 ± 1.22 (3.71–4.66)	4.14 ± 2.13 (3.05–4.69)	4.09 ± 1.85 (3.02–4.46)	4.12 ± 2.10 (3.37–5.00)
RT Knee—Flexion	2.59 ± 0.90 (2.00–2.70)	2.66 ± 0.90 (2.00–2.70)	2.65 ± 1.01 (1.99–2.77)	1.76 ± 1.05 (0.97–1.78)	1.67 ± 1.07 (1.05–1.89)	1.58 ± 1.10 (0.86–1.71)
RT Ankle—Abduktion	2.90 ± 1.62 (1.90–3.16)	2.97 ± 1.88 (1.59–3.05)	2.99 ± 1.97 (1.52–3.05)	2.10 ± 1.03 (1.46–2.26)	2.25 ± 1.10 (1.60–2.45)	2.05 ± 1.34 (1.10–2.15)
RT Ankle—Rotation	3.46 ± 1.10 (2.76–3.61)	3.58 ± 1.22 (2.68–3.63)	3.74 ± 1.30 (2.83–3.84)	5.78 ± 1.88 (4.96–6.42)	6.01 ± 2.13 (5.26–6.91)	6.03 ± 2.09 (5.36–6.98)
RT Ankle—Flexion	4.49 ± 1.27 (4.03–5.02)	4.50 ± 1.19 (4.09–5.01)	4.45 ± 1.30 (4.16–5.17)	9.08 ± 2.95 (8.15–10.43)	9.52 ± 2.71 (8.34–10.44)	9.49 ± 2.63 (8.27–10.31)
Pelvis—Flexion	1.69 ± 0.76 (1.17–1.76)	1.77 ± 0.79 (1.26–1.87)	1.81 ± 0.82 (1.28–1.91)	1.91 ± 1.11 (1.49–2.35)	1.98 ± 1.29 (1.47–2.47)	2.07 ± 1.34 (1.33–2.37)
Pelvis—Obliquity	2.52 ± 2.83 (0.68–2.88)	2.60 ± 3.02 (0.49–2.83)	2.57 ± 3.00 (0.51–2.83)	1.02 ± 0.60 (0.56–1.02)	1.02 ± 0.68 (0.48–1.01)	0.96 ± 0.65 (0.54–1.05)
Pelvis—Rotation	x	x	x	1.40 ± 1.21 (0.51–1.44)	1.42 ± 1.26 (0.36–1.34)	1.38 ± 1.17 (0.55–1.45)

**Table 3:** Results of the paired *t*-test for RMSE and ROME of every joint and all three sections. Bold values indicate non-significant differences between condition 1 and condition 2.

	RMSE			ROME		
	A	B	C	A	B	C
	*p*-Value	*p*-Value	*p*-Value	*p*-Value	*p*-Value	*p*-Value
LT Hip—Abduction	<0.001	<0.001	<0.001	<0.001	<0.001	<0.001
LT Hip—Rotation	<0.001	<0.001	<0.001	<0.001	<0.001	<0.001
LT Hip—Flexion	<0.001	<0.001	<0.001	0.004	0.011	0.006
LT Knee—Abduction	<0.001	<0.001	<0.001	0.002	<0.001	0.005
LT Knee—Rotation	<0.001	<0.001	<0.001	<0.001	<0.001	<0.001
LT Knee—Flexion	<0.001	<0.001	<0.001	<0.001	<0.001	<0.001
LT Ankle—Abduction	<0.001	<0.001	<0.001	<0.001	0.001	<0.001
LT Ankle—Rotation	<0.001	<0.001	<0.001	<0.001	<0.001	<0.001
LT Ankle—Flexion	<0.001	<0.001	<0.001	<0.001	<0.001	<0.001
RT Hip—Abduction	<0.001	<0.001	<0.001	<0.001	<0.001	<0.001
RT Hip—Rotation	<0.001	<0.001	<0.001	<0.001	<0.001	<0.001
RT Hip—Flexion	<0.001	<0.001	<0.001	**0.081**	<0.001	<0.001
RT Knee—Abduction	<0.001	<0.001	<0.001	<0.001	<0.001	<0.001
RT Knee—Rotation	<0.001	<0.001	<0.001	<0.001	<0.001	<0.001
RT Knee—Flexion	<0.001	<0.001	<0.001	<0.001	<0.001	<0.001
RT Ankle—Abduktion	<0.001	<0.001	<0.001	<0.001	<0.001	<0.001
RT Ankle—Rotation	<0.001	<0.001	<0.001	<0.001	<0.001	<0.001
RT Ankle—Flexion	<0.001	<0.001	<0.001	<0.001	<0.001	<0.001
Pelvis—Flexion	<0.001	<0.001	<0.001	<0.001	<0.001	<0.001
Pelvis—Obliquity	0.001	0.002	0.002	<0.001	<0.001	<0.001
Pelvis—Rotation	x	x	x	<0.001	<0.001	<0.001

**Table 4:** Mean intraclass correlation coefficient (ICC) values for the IMU system over all subjects ± SD; brackets contain 95% CI. A, B, C indicate beginning, middle, and end of the 6 min walk test.

	ICC ± SD (95% CI)		
	A	B	C
LT Hip—Abduction	0.92 ± 0.07 (0.90–0.96)	0.91 ± 0.07 (0.91–0.96)	0.92 ± 0.06 (0.91–0.96)
LT Hip—Rotation	0.75 ± 0.20 (0.70–0.86)	0.76 ± 0.18 (0.73–0.87)	0.76 ± 0.16 (0.72–0.84)
LT Hip—Flexion	0.98 ± 0.01 (0.98–0.99)	0.99 ± 0.01 (0.98–0.99)	0.99 ± 0.01 (0.99–0.99)
LT Knee—Abduction	0.57 ± 0.26 (0.53–0.73)	0.58 ± 0.27 (0.54–0.75)	0.57 ± 0.30 (0.52–0.75)
LT Knee—Rotation	0.69 ± 0.13 (0.65–0.75)	0.71 ± 0.13 (0.64–0.74)	0.71 ± 0.12 (0.66–0.76)
LT Knee—Flexion	0.98 ± 0.01 (0.97–0.98)	0.98 ± 0.01 (0.98–0.99)	0.98 ± 0.01 (0.98–0.99)
LT Ankle—Abduction	0.79 ± 0.09 (0.75–0.81)	0.79 ± 0.10 (0.77–0.84)	0.80 ± 0.08 (0.78–0.84)
LT Ankle—Rotation	0.82 ± 0.06 (0.81–0.86)	0.84 ± 0.07 (0.82–0.88)	0.85 ± 0.05 (0.85–0.89)
LT Ankle—Flexion	0.94 ± 0.02 (0.94–0.96)	0.94 ± 0.03 (0.94–0.96)	0.94 ± 0.03 (0.94–0.96)
RT Hip—Abduction	0.93 ± 0.05 (0.92–0.97)	0.92 ± 0.06 (0.91–0.96)	0.91 ± 0.07 (0.91–0.97)
RT Hip—Rotation	0.76 ± 0.20 (0.75–0.90)	0.76 ± 0.20 (0.74–0.90)	0.75 ± 0.22 (0.75–0.92)
RT Hip—Flexion	0.99 ± 0.01 (0.98–0.99)	0.98 ± 0.01 (0.98–0.99)	0.98 ± 0.01 (0.98–0.99)
RT Knee—Abduction	0.56 ± 0.34 (0.56–0.83)	0.56 ± 0.35 (0.57–0.84)	0.56 ± 0.34 (0.52–0.78)
RT Knee—Rotation	0.69 ± 0.14 (0.67–0.78)	0.69 ± 0.14 (0.64–0.75)	0.68 ± 0.16 (0.64–0.76)
RT Knee—Flexion	0.98 ± 0.01 (0.98–0.99)	0.98 ± 0.01 (0.98–0.99)	0.98 ± 0.01 (0.98–0.99)
RT Ankle—Abduktion	0.76 ± 0.13 (0.75–0.85)	0.77 ± 0.13 (0.76–0.86)	0.76 ± 0.16 (0.75–0.87)
RT Ankle—Rotation	0.85 ± 0.05 (0.83–0.87)	0.86 ± 0.05 (0.85–0.89)	0.86 ± 0.06 (0.84–0.88)
RT Ankle—Flexion	0.94 ± 0.02 (0.94–0.95)	0.95 ± 0.02 (0.94–0.96)	0.95 ± 0.03 (0.93–0.96)
Pelvis—Flexion	0.90 ± 0.06 (0.89–0.94)	0.90 ± 0.07 (0.89–0.95)	0.90 ± 0.08 (0.90–0.96)
Pelvis—Obliquity	0.52 ± 0.19 (0.50–0.64)	0.52 ± 0.20 (0.51–0.67)	0.52 ± 0.23 (0.47–0.65)
Pelvis—Rotation	0.82 ± 0.12 (0.82–0.91)	0.81 ± 0.12 (0.79–0.88)	0.78 ± 0.15 (0.79–0.91)

**Figure A1:**
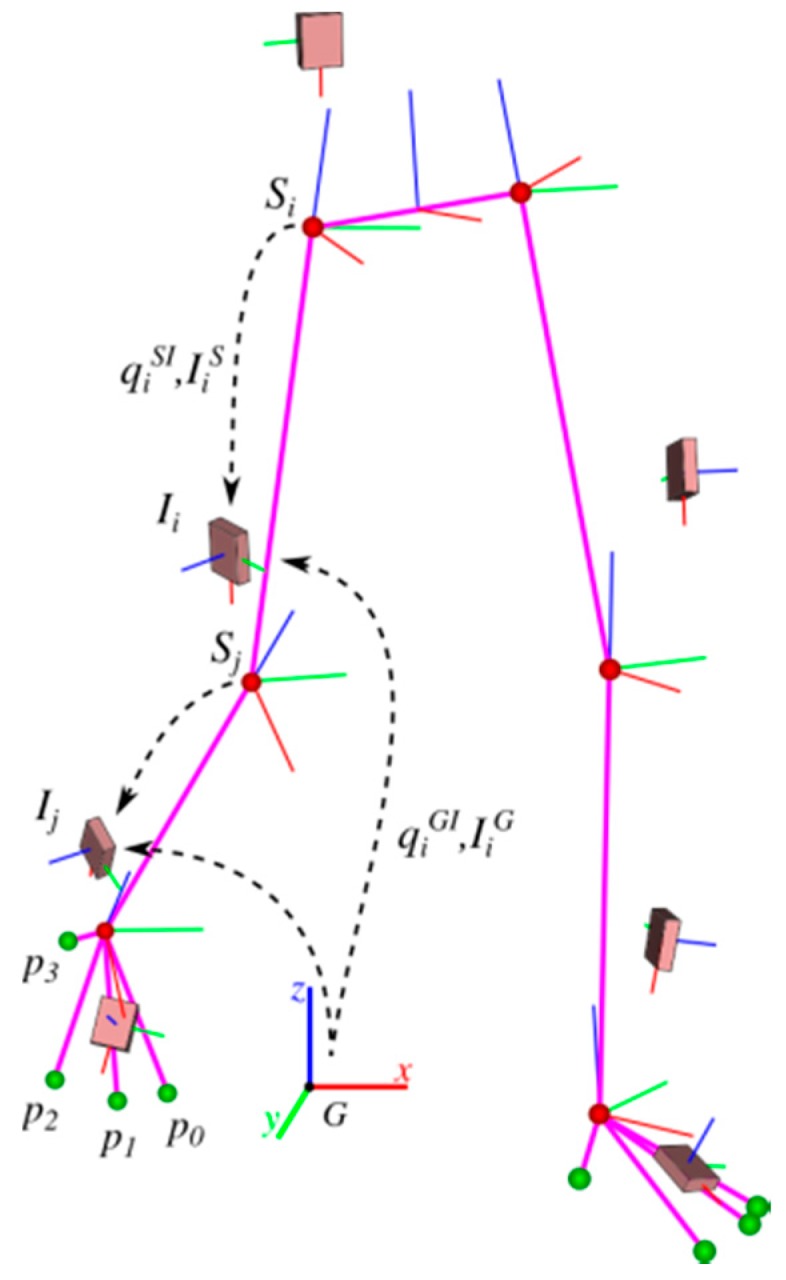
Biomechanical model of the lower body with segments (magenta lines), joint centers (red spheres), four ground contact points on each foot (green spheres), IMU placement, and involved coordinate frames. A technical coordinate system is associated to each IMU (I). The segment coordinate systems (S) are drawn at the proximal ends of the segments. The six DoF transformations, each in terms of an orientation (quaternion) $q^{SI}$ and a translation $I^{S}$, between the IMU coordinate frames and the associated segment coordinate frames are called IMU-to-segment calibrations. The symbol G denotes the global coordinate system. The figure has been taken from [14].

**Figure A3:**
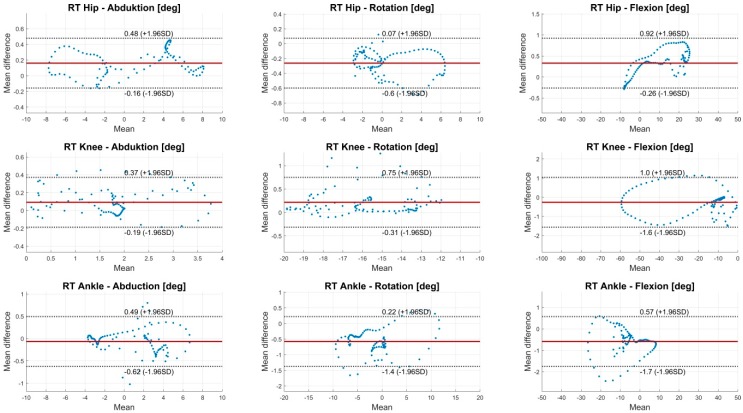
BA diagrams of 3D joint rotations of hip, knee and ankle of the right side for condition 1. The solid line indicates the mean difference. The dashed lines indicate the LoA (95% CI of the mean difference).

**Figure A7:**
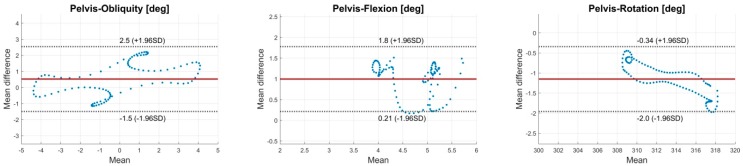
BA diagrams of 3D joint rotations of the pelvis for condition 2. The solid line indicates the mean difference. The dashed lines indicate the LoA (95% CI) of the mean difference.

## Appendix A

The here presented sensor fusion method for segment kinematics estimation is a slightly modified version of [14], which itself was based on [2]. Note that the contribution of [14] was the extension of the best performing extended Kalman filter (EKF)-based segment orientation estimation method of [2] with global translation estimation via ground contact estimation. For this study, the method of [14] was used while omitting magnetometer measurements (i.e., skipping measurement updates with the magnetometer data). Moreover, it was extended with online accelerometer bias estimation as proposed in [11,27] and by using an IEKF instead of an EKF [37]. For the reader’s convenience, the resulting method is detailed in the following. For further information, please refer to the abovementioned publications.

### Appendix A.1. Biomechanical Model

The biomechanical model is a stick-figure-like model with rigid segments. Each segment $S_{i}\in S$ has an associated segment coordinate frame localized in the global position-less coordinate system G. The latter has z pointing up (opposing gravity). The global six DOF pose of the i-th segment is represented by the unit quaternion $q^{GS}$ (denoting the orientation between G and S) and the position vector $S^{G}$ (denoting the position of frame S in G). Furthermore, each segment $S_{i}$ defines a set of points $P^{S_{i}}$ which are represented in the segment coordinate system and are used for joint definitions: a joint $J_{k}\in J$ defines which points from which segments are connected. Hence, a joint is a quadruple (i,k,j,l), meaning that $p_{k}\in P^{S_{i}}$ is connected to $p_{l}\in P^{S_{j}}$ in the global frame. Additionally, each segment $S_{i}$ has an IMU $I_{i}$ attached, with the rigid transformation ${\left\{q^{SI},\;I^{S}\right\}}_{i}$. This transformation is referred to as IMU-to-segment (I2S) calibration. The full model of the lower body is depicted in Figure A1.

### Appendix A.2. Segment Kinematics Estimation Method

The goal is to estimate the segment kinematics by fusing information from the gyroscope and accelerometer measurements with assumptions from a motion model as well as from the biomechanical model. This is realized through an IEKF and an IMU-centered state-space model, i.e., the IMU kinematics are estimated and the segment kinematics are derived from these using the following equations:

$$\;q^{GS}=q^{GI}⊙q^{IS}$$

$$\;S^{G}=I^{G}-R(q^{GS})I^{S},$$

where ⊙ denotes the quaternion multiplication, R(⋅) converts a quaternion to the corresponding rotation matrix and the I2S calibration is assumed known. Note, in [2], the IMU-centered state-space model outperformed the segment-centered state-space model for the considered tasks of magnetometer-free human motion tracking, which justifies this choice.

Hence, the state contains the following IMU-centered kinematic parameters of all *n* segments of the considered biomechanical model at time *t*:

$$x_{t}={\left({\left\{q^{GI},\;\omega_{I}^{GI},\;I^{G},\;{\dot{I}}^{G},\;{\ddot{I}}^{G},\;b^{a}\right\}}_{i=0}^{n-1}\right)}_{t}^{T},$$

where $q^{GI}$ is the orientation of the IMU wrt. the global frame, $\omega_{I}^{GI}$ is the rotational velocity of the IMU relative to the global frame, expressed in the IMU frame, $I^{G}$ is the IMU position in the global frame, and ${\dot{I}}^{G}$ and ${\ddot{I}}^{G}$ are the corresponding linear velocity and acceleration. The symbol $b^{a}$ denotes the accelerometer bias, represented in the IMU frame.

The motion model (used in the IEKF time update) assumes constant angular velocity (with noise in angular acceleration), constant linear acceleration (with noise in jerk) and a random walk for the accelerometer bias. Hence, after *T* seconds, the prediction for the state variables is

$$x_{t+T}=\left[\begin{array}{c} q_{i,t}^{GI}⊙\exp\left(\frac{T}{2}\omega_{I,i,t}^{GI}\right)\\ \omega_{I,i,t}^{GI}+T{\hat{\delta }}_{t}^{\omega }\\ I_{i,t}^{G}+T{\dot{I}}_{i,t}^{G}+\frac{T^{2}}{2}{\ddot{I}}_{i,t}^{G}\\ {\dot{I}}_{i,t}^{G}+T{\ddot{I}}_{i,t}^{G}\\ {\ddot{I}}_{i,t}^{G}+T{\hat{\delta }}_{t}^{a}\\ b_{i,t}^{a}+{\hat{\delta }}^{b}\\ \vdots \end{array}\right],$$

where exp(⋅) is the quaternion exponential and ${\hat{\delta }}^{\omega }\sim N\left(0,\;{\hat{\Sigma }}^{\omega }\right)$, ${\hat{\delta }}^{a}\sim N\left(0,\;{\hat{\Sigma }}^{a}\right)$ and ${\hat{\delta }}^{b}\sim N\left(0,\;{\hat{\Sigma }}^{b}\right)$ are Gaussian white process noises. The vertical dots indicate that these variables are given for each IMU i∈{0,…,n−1}.

The (implicitly formulated) measurement models for the inertial measurements, i.e., the acceleration $y_{i,t}^{a}$ and the rotational velocity $y_{i,t}^{\omega }$, are

$$0=y_{i,t}^{a}-b_{i,t}^{a}-R{\left(q_{i,t}^{GI}\right)}^{T}\left[{\ddot{I}}_{i,t}^{G}-g^{G}\right]-\delta_{i}^{a},$$

and

$$0=\;y_{i,t}^{\omega }-\;\omega_{I,i,t}^{GI}-\delta_{i}^{\omega },$$

where ${\left(\cdot \right)}^{T}$ is the matrix transpose, $g^{G}$ denotes acceleration due to gravity in the global system, and $\delta^{a}\sim N\left(0,\;\Sigma^{a}\right)$ as well as $\delta^{\omega }\sim N\left(0,\;\Sigma^{\omega }\right)$ are Gaussian white measurement noises.

Biomechanical model constraints, i.e., the fact that segments are connected at the joints, are also modeled as noisy measurements (hence, soft constraints), where the noises account for inaccuracies of the assumed biomechanical model. Let joint (i,k,j,l) connect point $p_{k}\in P^{S_{i}}$ with $p_{l}\in P^{S_{j}}$ in the global frame, the measurement model is

$$\begin{array}{l} 0=p_{k}^{G}-p_{l}^{G}-\delta^{p}\\ 0=S_{i}^{G}+R\left(q_{i}^{GS}\right)p_{k}^{S_{i}}-\left[S_{j}^{G}+R\left(q_{j}^{GS}\right)p_{l}^{S_{j}}\right]-\delta^{p}\\ 0=\;S_{i}^{G}+R\left(q_{i}^{GI}⊙q_{i}^{IS}\right)p_{k}^{S_{i}}-\left[S_{j}^{G}+R\left(q_{j}^{GI}⊙q_{j}^{IS}\right)p_{l}^{S_{j}}\right]-\delta^{p}\\ 0=I_{i}^{G}+\;R\left(q_{i}^{GI}⊙q_{i}^{IS}\right)\left[p_{k}^{S_{i}}-I_{i}^{S}\right]-I_{j}^{G}-\;R\left(q_{j}^{GI}⊙q_{j}^{IS}\right)\left[p_{l}^{S_{j}}-I_{j}^{S}\right]-\delta^{p}, \end{array}$$

where $\delta^{p}\sim N\left(0,\;\Sigma^{p}\right)$ denotes Gaussian white measurement noise.

Environmental constraints in terms of ground contacts are also incorporated into the estimation as noisy measurements. For this, in each time step, probabilistic ground contact estimations are carried out in parallel to the kinematics estimation for a set of potential ground contact points $P^{c}\subset \overset{n-1}{\underset{i=0}{⋃}}P^{S_{i}}$ (cf. Figure A1). For each of these points, ground contact probabilities are calculated, given the point height and velocity as obtained from the kinematics estimation. Let $p_{k}\in P^{S_{i}}$ and also $p_{k}\in P^{C}$, then

$$\;u_{k}={\left(p_{k}^{G}\right)}_{z}={\left[I_{i}^{G}+\;R\left(q_{i}^{GI}⊙q_{i}^{IS}\right)\left[p_{k}-I_{i}^{S}\right]\right]}_{z}$$

is the estimated global point height, and

$$\;y_{k}={\dot{I}}_{i}^{G}+R\left(q_{i}^{GI}\right)S\left(\omega_{I,i}^{GI}\right)\left[R\left(q_{i}^{IS}\right)\left(p_{k}-I_{i}^{S}\right)\right]\;$$

is the estimated global point velocity. Here, S(⋅) denotes the skew-symmetric matrix of a vector.

For information fusion, a binary discrete Bayes filter [38] is used for each potential contact point. Assuming the prediction being independent of the previous state, given a random variable $X_{t}=\text{\{}x_{1}\;∶=contact\;|\;x_{2}:=\;no\;contact\}$ the Bayes filter simplifies to

$$p\left(X_{t}=x_{1}\right)=\eta p\left(X_{t-1}=x_{1}|u_{k}\right)p\left(y_{k}|X_{t}=x_{1}\right),$$

with

$$\eta ={\left(1-p\left(X_{t-1}=x_{1}|u_{k}\right)+2p\left(y_{k}|X_{t}=x_{1}\right)p\left(X_{t-1}=x_{1}|u_{k}\right)+p\left(y_{k}|X_{t}=x_{1}\right)\right)}^{-1}.$$

The probabilities are obtained as follows:

$$p(X_{t-1}=x_{1}|u_{k})=\;1-sig_{\left\{0,5,0.9,60,0.05\right\}}\left(u_{k}\right),$$

for the prediction, and

$$p(y_{k}|X_{t}=x_{1})=1-sig_{\left\{0.5,\;0.95,\;0.9,\;0.7\right\}}\left(y_{k}\right),$$

as measurement using the sigmoid function

$$sig_{\left\{c,m,s,o\right\}}\left(x\right)=c+\frac{m}{2}\tanh\left(s\left(x-o\right)\right).$$

Here, the parameters for the sigmoid function were empirically determined. For each ground contact point, these probabilities are calculated in every time step. If the largest ground contact probability exceeds a threshold $p_{th}$, then two soft constraints (in terms of measurement updates) are applied to the corresponding point $p_{k}$ inside the kinematics estimation filter: a zero-velocity update

$$\;0=\;{\dot{I}}_{i}^{G}+R\left(q_{i}^{GI}\right)S\left(\omega_{I,i}^{GI}\right)\left[R\left(q_{i}^{IS}\right)\left(p_{k}-I_{i}^{S}\right)\right]+\delta^{\dot{p}}$$

and, to prevent a drift in height, a zero-plane update

$$\;0={\left[I_{i}^{G}+\;R\left(q_{i}^{GI}⊙q_{i}^{IS}\right)\left[p_{k}-I_{i}^{S}\right]\right]}_{z}+\delta^{z}$$

with $\delta^{\dot{p}}\sim N\left(0,\;\Sigma^{\dot{p}}\right)$ and $\delta^{z}\sim N\left(0,\;\Sigma^{z}\right)$ being Gaussian white measurement noises. If the threshold was not exceeded, a zero-plane update is applied to the lowest ground contact point only if this has a negative height. Otherwise no additional measurement update is conducted. Obviously, in the current state, the method assumes one plane. Table A1 contains all previously introduced noises and parameters.

**Table A1:** Noise and parameter settings for the presented kinematics estimation method.

Parameter	$\;{\hat{\Sigma }}^{\omega }$	$\;{\hat{\Sigma }}^{a}$	$\;{\hat{\Sigma }}^{b}$	$\;\Sigma^{\omega }$	$\;\Sigma^{a}$	$\;\Sigma^{p}$	$\;\Sigma^{\dot{p}}$	$\;\Sigma^{z}$	$\;p_{th}$
Value	$\;10^{5}\times I_{3\times 3}$	$\;3\times 10^{5}\times I_{3\times 3}$	$\;10^{-8}\times I_{3\times 3}$	$\;10^{-3}\times I_{3\times 3}$	$\;10^{-2}\times I_{3\times 3}$	$\;10^{-4}\times I_{3\times 3}$	$\;10^{-2}\times I_{3\times 3}$	10^−5^	0.7
